# Bilateral Asymmetry of Spatiotemporal Running Gait Parameters in U14 Athletes at Different Speeds

**DOI:** 10.3390/sports12050117

**Published:** 2024-04-27

**Authors:** Antonio Cartón-Llorente, Silvia Cardiel-Sánchez, Alejandro Molina-Molina, Andrés Ráfales-Perucha, Alberto Rubio-Peirotén

**Affiliations:** Campus Universitario, Universidad San Jorge, Autov A23 km 299, Villanueva de Gállego, 50830 Zaragoza, Spain; acarton@usj.es (A.C.-L.); scardiels@usj.es (S.C.-S.); arafales@usj.es (A.R.-P.); arubio@usj.es (A.R.-P.)

**Keywords:** adolescent, running kinematics, track and field, biomechanics, wearables

## Abstract

The assessment of leg asymmetries is gaining scientific interest due to its potential impact on performance and injury development. Athletes around puberty exhibit increased gait variability due to a non-established running pattern. This study aims to describe the asymmetries in the spatiotemporal running parameters in developmentally aged athletes. Forty athletes under 14 (U14) (22 females and 18 males) were assessed running on a treadmill at constant speeds of 12 and 14 km·h^−1^ for 3 min. Step length, step frequency, along with contact (CT) and flight time, both in absolute values and as a percentage of step time, were recorded using a RunScribe sensor attached to the laces of each shoe. U14 runners exhibited high bilateral symmetry in the spatiotemporal parameters of running, with mean asymmetry values (1–5.7%) lower than the intra-limb coefficient of variation (1.7–9.6%). Furthermore, bilateral asymmetries did not vary between the two speeds. An individual-based interpretation of asymmetries identified subjects with consistent asymmetries at both speeds, particularly in terms of CT and contact ratio (%, CT/step time). This study confirms the high symmetry of pubertal runners and paves the way for the application of portable running assessment technology to detect asymmetries on an individual basis.

## 1. Introduction

The assessment of functional asymmetries has garnered significant interest in the sports science community in the last few years, both in individual and team sports [1,2,3,4]. This heightened attention is attributed to the growing awareness of the link between limb imbalance and performance losses [5], as well as their potential association with an increased risk of sports-related injuries [6,7,8].

In the context of sport, bilateral asymmetry refers to the disparity in the function or performance of one limb relative to the other [9]. An overall threshold of 10–15% has previously been suggested to identify “abnormal” interlimb asymmetry [10]. However, the evidence for the validity of such arbitrary thresholds is weak, and generalizing their use should be avoided. Instead, an individualized approach to delineate asymmetry might be critical for enhancing robust calculation methods and establishing appropriate thresholds across different samples and methodologies, facilitating the derivation of well-founded conclusions [11].

Given the wide array of protocols previously employed to measure asymmetries, professionals should assess which one is most suitable for their athletes based on an analysis of the sport’s specific requirements, the athletes’ characteristics, and the cost-effectiveness of the test. Traditionally, inter-limb asymmetry assessment has focused on quantifying strength differences in mono and bipodal tasks, utilizing laboratory tests such as dynamometry or force platforms [12,13].

In recent years, there has been a shift towards incorporating alternative methods to assess functional asymmetries in a more practical way. As a result, jumping tests have become the mainstream proxy for functional strength as they mimic relevant movement patterns (i.e., the triple extension of lower limb joints) observed in sport [1,14,15]. In team sports such as soccer, asymmetries have been studied in sport-specific motor tasks, the 10 m sprint, changes in direction in dribbling or running [3]. Or in basketball with movements like jumping or changing direction [4]. This shift aims to move the evaluation of asymmetries into more ecological, sport-specific and time-efficient environments.

In this context, it seems appropriate to explore the bilateral symmetry of runners during the task itself. Previous research has revealed that the behavior of the legs during running is highly symmetrical, with low to moderate (<5%) mechanical asymmetries for spatiotemporal variables [16,17,18]. However, when assessing kinetics and spring-mass model properties, asymmetry scores increase up to 8% and 20%, respectively [19]. Of note, elite runners appear to have lower percentages of asymmetry compared to amateur and novice runners, while higher running speed may be associated with greater gait symmetry [2,20]. However, previous research findings have been inconsistent in the latter sense [19,21].

It is worth noting that a rigorous assessment of intrasubject variability is essential for an accurate interpretation of bilateral asymmetries in running [22]. Thus, the intra-limb variability must be less than the inter-limb difference for the bilateral asymmetry to be deemed significant. In this regard, previous research [23] found significant asymmetries in less than half of the kinematic variables (such as, step length [SL], step frequency [SF], contact time [CT] and flight time [FT]), but pointed out that the variables showing significant asymmetry were specific of the athlete.

Among runners of all ages, adolescents tend to show more variability and asymmetry in their running gait patterns, indicating a lack of mature running gait during the ongoing growth phase [24,25]. In particular, pubertal runners (i.e., around their peak height velocity) may need to adapt their running patterns due to the rapid changes in leg length and muscle strength, making it a sensitive age for the development of functional asymmetries. Early detection of performance-limiting factors could be helpful in targeting motor learning programs to improve neuromuscular control during running [26]. Therefore, the aims of the present study were twofold: (i) to provide an accurate interpretation of running kinematic asymmetries in under-14 (U14) athletes, and (ii) to compare their inter-limb asymmetry values at two running speeds: 12 and 14 km·h^−1^. The authors hypothesized that under-14 athletes will exhibit a fair inter-limb symmetry running at 12 and 14 km·h^−1^. It was also hypothesized that higher velocities may amplify biomechanical discrepancies in running gait, potentially due to the challenges in maintaining symmetrical movement patterns under increased mechanical stress during rapid locomotion.

## 2. Materials and Methods

### 2.1. Study Desing

This study followed a cross-sectional design. All U14 athletes and their parents received detailed information regarding the potential risks associated with the test, and written informed consent was obtained in accordance with the ethical principles described in the Declaration of Helsinki. The study was carried out after approval by the Clinical Research Ethics Committee of the Government of Aragón (PI23/223, CEICA, Spain).

### 2.2. Subjects

Forty U14 track and field athletes (22 females and 18 males) voluntarily participated in this study (chronological age: 12.3 ± 0.6 years; height: 154.3 ± 7.5 cm; body mass: 43.0 ± 8.3 kg; body mass index [BMI]: 17.8 ± 2.2). All participants met the inclusion criteria: [i] born in 2011 or 2012, [ii] previous experience running on a treadmill (at least three times in the last four weeks), [iii] discrepancy in leg length less than 3 cm, and [iv] not having pain or lower limb injuries in the four weeks prior to data collection that would require them to stop training for more than two weeks.

### 2.3. Procedures

On the selected day, participant’s sex, date of birth, and anthropometric measures (body mass, sitting and standing height, leg length and ankle dorsiflexion) were recorded using a stadiometer (±0.1 cm; SECA, 214, SECA, Hamburg, Germany), a digital scale (±0.1 kg; BC-601, Tanita, IL, USA), and a digital inclinometer (microFET3; Hogan Health Industries, Salt Lake City, UT, USA), according to the protocols recommended by the International Society for the Advancement of Kinanthropometry (ISAK) [27]. Peak Height Velocity (PHV) was then estimated based on the predictive equation described by Mirwald et al. [28] (standard error of measurement [SEM] = 0.592), and maturity offset was calculated by subtracting the PHV from the chronological age at the time of measurement.

#### Running Assessment

Prior to the recording, the subjects performed an 8 min accommodation run on the treadmill increasing speed by 0.5 km/h^−1^ every minute from 8 to 12 km/h^−1^ [29]. After that, subjects completed two 3 min runs, at 12 and 14 km/h^−1^, with a 5 min rest between them. All runs were conducted on a motorized treadmill (HP cosmos Pulsar 4P; HP cosmos Sports & Medical, Gmbh, Nußdorf, Germany) with a slope of 0%. The kinematic and kinetic data for each leg were recorded using a pair of RunsScribe Red pods (Scribe Lab Inc., San Francisco, CA, USA) attached to the right (unit 1) and left (unit 2) shoelaces, in accordance with the system manufacturer’s recommendations for all participants. This nine-axis IMU system (three-axis gyroscope, three-axis accelerometer, three-axis magnetometer) captures time-motion variables with a sampling rate of 500 Hz (accuracy of 0.002 s), has been previously validated for the assessment of running gait [30]. Running spatiotemporal parameters from the central 2 min were collected for CT (s), which represents the duration from foot contact with the ground to toe lift-off; FT (s), denoting the time from toe-off to the initial ground contact of consecutive footfalls (e.g., right–left); SL (m), indicating the distance the treadmill belt moves from toe-off to initial ground contact in successive steps from forefoot to forefoot; and SF (steps per minute, spm), representing the number of ground contact events per minute. To stress the importance of interpreting contact and flight times relative to individual’s step time, contact ratio (CR, %) and flight ratio (FR, %) were also collected from the developers website (https://dashboard.runscribe.com/runs, accessed on 20 March 2024) into the .csv file. Then, data were imported into Excel^®^ (v. 2401, Microsoft, Inc., Redmond, WA, USA) and further analyzed.

### 2.4. Statistical Analysis

All statistical analyses were conducted using SPSS software (Version 29.0, SPSS Inc., Chicago, IL, USA). Normality assessment was performed using the Shapiro-Wilk test. Descriptive data are presented as mean and standard deviation (SD). Within-session reliability was calculated on an individual basis using the coefficient of variation (CV = SD/mean × 100) for absolute reliability, along with a two-way random intraclass correlation coefficient (ICC) for relative reliability, with the complete agreement and 95% confidence intervals. Interpretation of the ICC values followed the guidelines provided by Koo et al. [31]. A CV below 10% was deemed as an acceptable criterion for reliability following previous research [22].

Bilateral asymmetries were calculated according to the existing guidelines for expressing percentage differences based on unilateral tests [32]. Therefore, the following calculation formula was selected to compare the data reported by both RunScribe pods:

(1) $$BA\;\left(\%\right)=\frac{100}{Max\;value\times Min\;value\times -1}+100$$

To interpret bilateral asymmetries, only those parameters that showed bilateral asymmetries greater than the intra-limb variability were considered to be significantly asymmetrical [23]. In addition, paired sample t-tests were performed to identify significant differences between the right and left limb values and between bilateral asymmetries at speeds of 12 and 14 km·h^−1^, for each relevant parameter. Cohen effect sizes, with a 95% confidence interval, were also computed to quantify the magnitude of pairwise comparisons and was interpreted as trivial (<0.2), small (>0.2), moderate (>0.5), or large (>0.8) [33]. Finally, to explore the behavior of bilateral asymmetries as a function of speed, pairwise comparisons were performed between speeds of 12 and 14 km·h^−1^ for each variable studied. Statistical significance was inferred from *p* < 0.05.

## 3. Results

Descriptive characteristics of the participants are shown in Table 1. For the selected running spatiotemporal variables, Table 2 provides information on within-session reliability and bilateral asymmetry. All examined parameters showed good to excellent reliability (ICC: 0.78 to 0.99) and acceptable variability (CV ≤ 9.7%), except for the FT at 12 km·h^−1^ (CV = 10.4%).

**Figure 1:**
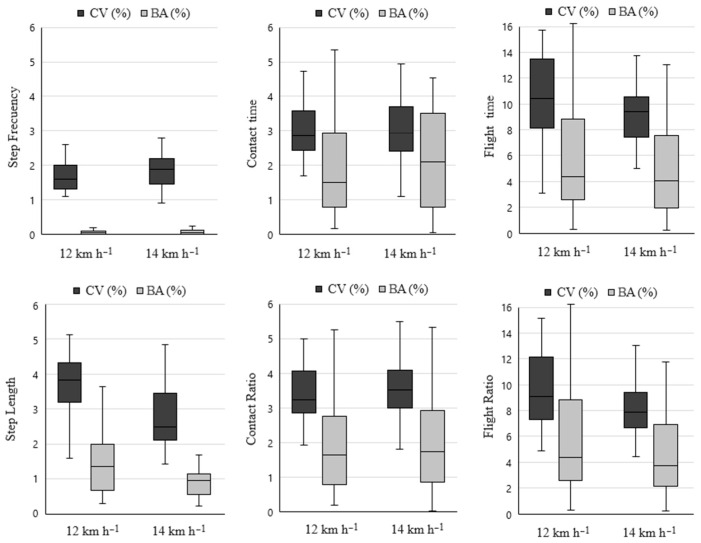
Box plots of the intra-limb variation (coefficient of variation) and inter-limb asymmetries (bilateral asymmetries) for the selected spatiotemporal running gait variables at 12 and 14 km·h^−1^.

Bilateral asymmetry analysis did not reveal any significant differences between right and left mean values for any of the variables examined. Mean inter-limb asymmetries ranged from 1.0 to 5.7. However, all variables displayed a higher mean intra-limb variability than bilateral asymmetry (Figure 1).

**Figure 2:**
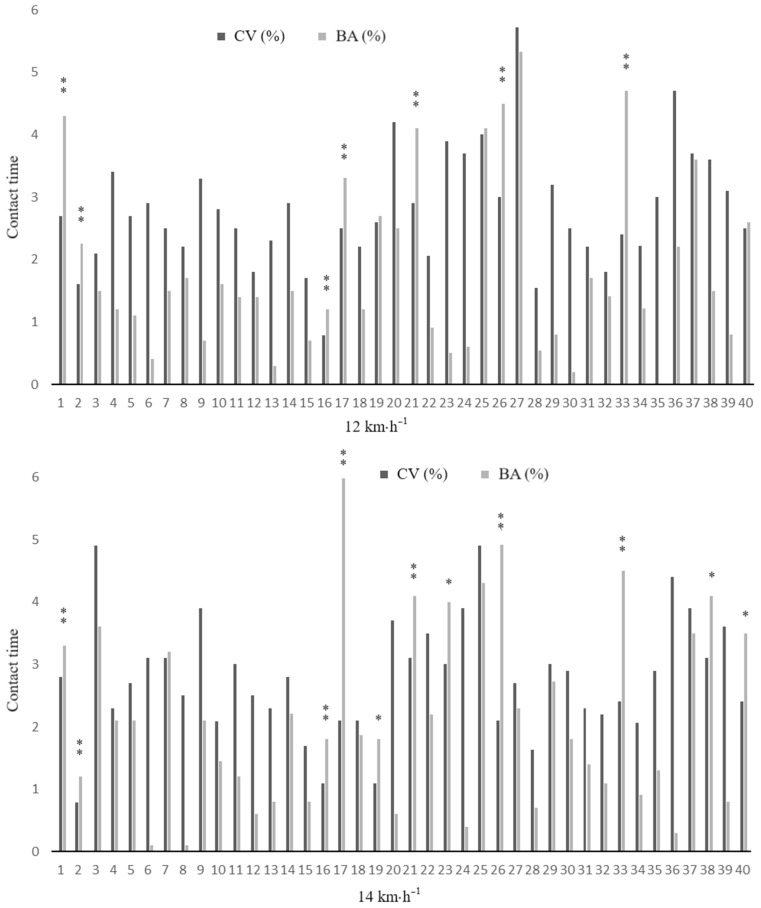
Individual coefficient of variation and bilateral asymmetry data for contact time at 12 and 14 km·h^−1^. * denotes meaningful asymmetry (BA% > CV), and ** meaningful asymmetry (BA% > CV) and consistency at both velocities (12 and 14 km/h).

**Figure 3:**
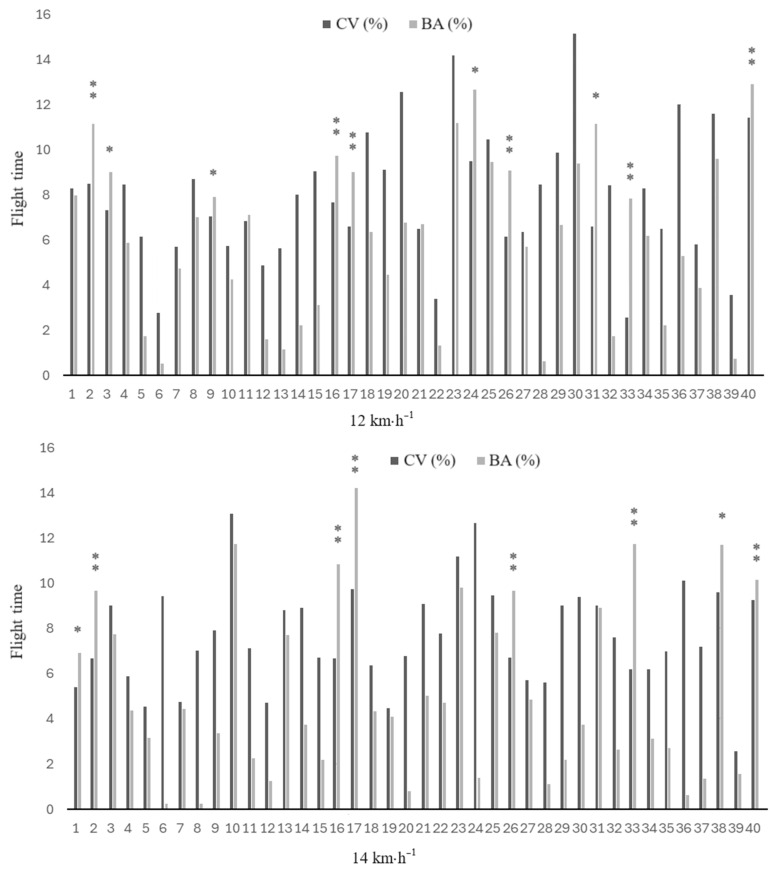
Individual coefficient of variation and bilateral asymmetry data for flight time at 12 and 14 km·h^−1^. * denotes meaningful asymmetry (BA% > CV), and ** meaningful asymmetry (BA% > CV) and consistency at both velocities (12 and 14 km/h).

**Figure 4:**
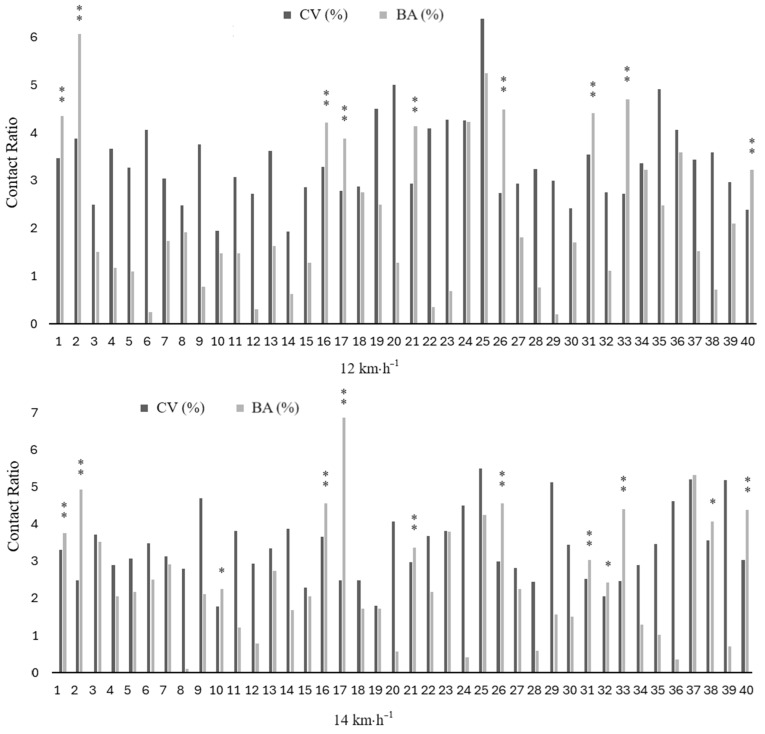
Individual coefficient of variation and bilateral asymmetry data for contact ratio at 12 and 14 km·h^−1^. * denotes meaningful asymmetry (BA% > CV), and ** meaningful asymmetry (BA% > CV) and consistency at both velocities (12 and 14 km/h).

Pairwise comparisons of mean values, coefficients of variation and percentages of bilateral asymmetry between running speeds (12 and 14 km·h^−1^) are shown in Table 3. Although all spatiotemporal variables showed differences between both speeds (*p* ≤ 0.013; ES: 0.6 to 3.0), bilateral asymmetries did not differ significantly between speeds. Individual inter-limb discrepancies for CT, FT and CR at 12 and 14 km·h^−1^ are illustrated in Figure 2, Figure 3 and Figure 4.

## 4. Discussion

The objectives of the investigation were to examine spatiotemporal asymmetries in running gait among U14 athletes and to determine whether disparities in inter-limb asymmetry scores existed at speeds of 12 and 14 km·h^−1^. The main findings of the present study were: (i) U14 runners exhibited bilateral symmetry for spatiotemporal parameters of running at different speeds, with asymmetry percentages ranging from 1.0 to 5.7; (ii) bilateral asymmetries showed no significant differences between 12 and 14 km·h^−1^; and (iii) the individualized analysis was able to identify subjects with significant inter-limb differences for CT, FT, and CR.

SF, SL, CT and CR showed high absolute and relative reliability (CV ≤ 3.7%; ICC: 0.91 to 0.96), indicating that the data can be interpreted with confidence for further analysis [34]. On the other hand, FT and FR exhibited wide CV ranging from 8 to 10.4%, especially at low speeds, which hinders their use for asymmetry identification [22]. In this regard, previous studies have evaluated the reliability of the running kinematic parameters reported by the RunScribe system at different speeds and across various terrains [35,36], demonstrating similar coefficients of variation for the analyzed variables (CV < 4%). Furthermore, the RunScribe system has demonstrated good accuracy for the running spatiotemporal parameters analyzed herein, with differences ≤ 3% compared to a gold standard method [37]. It is worth noting that the reliability and validity of this sensor for the assessment of other running variables, such as foot strike pattern, ground reaction forces, shock, impact, pronation degrees and its angular velocity during foot contact, has also been previously examined [30,38]. However, these novel metrics have shown wider CV (up to 36%) and only moderate reliability (ICC = 0.5–0.75) [36]. Current results for these novel metrics in the studied population are available in Table S1 in the Supplementary Materials.

Regarding the primary outcome of the study, U14 athletes showed high inter-limb symmetry in kinematic parameters while running at 12 and 14 km·h^−1^, with negligible asymmetries in SF (<1%), small in SL, CT and CR (≤2%), and fair in FT and FR (<6%). However, pairwise comparisons highlight the lack of significant differences between limbs for any of the variables studied. While certain groups have defined inter-limb differences as significant only when the asymmetry score surpasses 10% [39,40], alternative perspectives propose that asymmetry should exceed intra-limb variability to be deemed significant [22,23]. In the present work, all variables exhibited CV higher than bilateral asymmetry, highlighting the predominantly symmetric nature of the spatiotemporal parameters of running. In this direction, previous works confirmed that running kinematics are highly symmetrical, with low-to-moderate (<5%) bilateral asymmetries for spatiotemporal variables in indoor [16,18,24] and outdoor [17] settings. Despite sprinting may not be entirely comparable to the submaximal speeds evaluated in the present work, bilateral asymmetries found in these studies seem to be in line with the results reported hereabouts for SF, SL and FR, but are slightly higher for CT (~4%).

On the other hand, previous studies have found higher CT in the injured leg compared to the non-injured side in well-trained athletes with a history of running-related injuries [7], and loading rate was also greater in the injured leg for recreational runners with unilateral tibial stress fracture [39]. In addition, a higher loading rate on the preferred leg has been linked to increased stress on the Achilles tendon compared to the non-preferred leg in healthy recreational runners [19]. Thus, bilateral asymmetry during running has been considered a risk factor [40,41], although the evidence is inconsistent in this regard [8,42,43].

Individual comparisons at different speeds (i.e., 12 and 14 km·h^−1^) revealed significant differences for all spatiotemporal variables (*p* ≤ 0.013; ES: 0.6 to 3.0). However, bilateral asymmetries did not change across speeds. Previous research on the role of running speed on asymmetries is inconclusive. Results from studies by Girard and Jiang [21,44] are consistent with ours, showing no change in bilateral asymmetry across speeds (10, 12.5, 15, 17.5, 20, 22.5, and 25 km·h^−1^). In contrast, another study [2] found group-dependent differences in asymmetries across speeds ranging from 8 to 12 km·h^−1^. Specifically, competitive runners showed a linear reduction in asymmetries with increasing speed, whereas recreational and novice runners did not exhibit such a pattern. It should be noted that the differences in asymmetries found in this study mainly affected the FT. Given that the natural transition between walking and running occurs at approximately 7.2 km·h^−1^ [45], the differences found between athletes of different levels in the aforementioned study could be partially explained by the slow running speeds with which they were compared (i.e., 8, 9 vs. 10, 11 and 12 km·h^−1^). Indeed, Table 3 shows that the only CV that significantly decreased between 12 and 14 km·h^−1^ was for FT (*p* = 0.003; ES = 0.4). This could be explained by the limited FT required to maintain this speed, prompting us to select a somewhat more demanding pace when assessing asymmetries (i.e., 14 km·h^−1^).

Unlike walking, which is defined as an almost pendulum-like motion in which kinetic energy increases as potential energy decreases and vice versa, running adopts a motion similar to a ball bouncing off the ground, in which kinetic and potential energies are in phase [46]. In this sense, it has been suggested that the perfect phasing between the legs is achieved at higher speeds [2]. Furthermore, while gait patterns do not mature until 13 years of age, it takes at least until 15 year of age to control the complexity of running [25]. Therefore, it would be expected that U14 athletes would be particularly susceptible to show mechanical asymmetries during running among the different levels of runners. However, the findings of the current study confirm that even athletes around their peak height velocity, indicating an ongoing development of running motor control, exhibited consistent bilateral symmetry values across the assessed speeds.

Finally, given the large between-subject differences in bilateral asymmetries found in this study, we included individualized intra- and inter-limb data for the kinematic variables of interest (Figure 2, Figure 3 and Figure 4). The individualized analysis of asymmetries identified subjects with asymmetries at 12 and 14 km·h^−1^ for CT, FT, and CR. Some subjects exhibited asymmetries in CT but not in FT, or vice versa. These discrepancies in absolute values could be attributed to minor changes in SF throughout the running test. However, it is important to note that SF could vary during the running test in a natural intra-runner pattern, even when speed is controlled. When SF changes, the absolute values do not necessarily change by the same extent for both values (i.e., they are no longer complementary or in the same relationship to each other as they were before). This is one of the reasons why relative values such as CR and FR are useful for comparing findings when controlling for speed but not for SF. Moreover, the current findings have shown that CR is a highly reliable method for detecting asymmetries in individuals (Figure 4). In particular, the analysis of CR provides an interesting measure relative to the total step. This metric, analogous to Millet and Morin’s duty factor [47], provides a deeper insight into the overall running pattern than when spatiotemporal parameters are considered independently [48]. In this regard, a previous study [42] found significant asymmetries in the sprint running kinematic variables in more than half of the subjects analyzed (i.e., ≥11 out of 22 participants), and another study [49] confirmed the individual athlete approach as the most appropriate for identifying asymmetries. These results highlight that asymmetries must be interpreted individually for each athlete and each variable, and underscore the limitations of using arbitrary thresholds to identify significant differences between limbs.

Lastly, there are certain limitations that need to be acknowledged. While we used wearable sensors to assess running biomechanics, the running assessments took place indoors. Although the sensor parameters used have been previously validated using a gold standard [30], the device does not allow for verification as access to the raw signals is not available. Existing research suggests that treadmill running is broadly comparable to overground running [50], leading us to opt for an indoor setup to standardize measurements. Additionally, participants underwent a pre-test familiarization session on a separate day to minimize the accommodation effect to treadmill running.

Despite these limitations, the current findings underscore the potential of wearable sensors for the identification of kinematic asymmetries in pubertal athletes. In this sense, interpretations of the asymmetries should be cautious and contemplate the intra-limb variability in the runners.

## 5. Practical Implications

This study evidences the prevalence of high bilateral symmetry in spatiotemporal parameters of running among athletes around puberty and that the degree of asymmetry between limbs remains constant regardless of running speed. However, an individualized assessment revealed a subset of athletes with consistent asymmetries in both speeds assessed. These results underscore the potential of portable running assessment technology to identify individualized asymmetries, thereby contributing to a deeper understanding of the running mechanics of young athletes. Future research into the role that spatiotemporal running asymmetries may play on performance and the long-term development of running-related injuries could build on the insights provided hereby.

## Figures and Tables

**Table 1:** Descriptive data of the participants. (mean, ± SD; n, %).

Variable		
Sex	Male	18 (45%)
	Female	22 (55%)
Age (years)		12.2 ± 0.6
Height (cm)		155.68 ± 7.02
Body Mass (kg)		40.1 ± 7.0
BMI (kg/m^2^)		17.4 ± 1.7
Maturity Offset (years)		−0.67 ± 1.30
Ankle dorsiflexion (°)		45.0 ± 8.4
Leg length asymmetry (%)		0.8 ± 0.8

**Table 2:** Mean values (right and left), reliability data and mean bilateral asymmetry (%) for each kinematic variable at 12 and 14 km·h^−1^. Results of inter-limb paired t-tests for all parameters of interest at both speeds.

Speed	Variables	Leg	Mean (SD)	CV (%)	ICC (95% CI)	BA (%)	*p*-Value
**12 km·h^−1^**	SF (spm)	Right Left	177.4 ± 7.3 177.6 ± 7.3	1.7 1.7	0.96 (0.93–0.98) 0.94 (0.92–0.97)	0.1	0.712
	SL (cm)	Right Left	111.7 ± 5.0 112.0 ± 4.9	3.7 3.9	0.88 (0.78–0.94) 0.86 (0.83–0.91)	1.5	0.442
	CT (ms)	Right Left	248 ± 16 249 ± 15	3.0 2.9	0.92 (0.87–0.95) 0.92 (0.86–0.95)	2.0	0.311
	FT (ms)	Right Left	92 ± 21 91 ± 21	10.2 10.7	0.98 (0.96–0.99) 0.96 (0.92–0.98)	5.5	0.223
	CR (%)	Right Left	73.2 ± 5.6 73.5 ± 5.5	3.4 3.4	0.93 (0.88–0.96) 0.94 (0.89–0.97)	2.1	0.428
	FR (%)	Right Left	26.8 ± 5.6 26.5 ± 5.5	9.6 9.9	0.78 (0.56–0.88) 0.79 (0.60–0.86)	5.7	0.402
**14 km·h^−1^**	SF (spm)	Right Left	181.5 ± 8.5 181.4 ± 8.5	1.9 1.9	0.94 (0.88–0.97) 0.94 (0.92–0.98)	0.1	0.482
	SL (cm)	Right Left	127.3 ± 6.1 127.2 ± 6.4	3.5 3.5	0.91 (0.85–0.95) 0.90 (0.83–0.98)	1.0	0.575
	CT (ms)	Right Left	229 ± 14 229 ± 13	3.0 3.0	0.94 (0.88–0.97) 0.95 (0.93–0.98)	2.0	0.980
	FT (ms)	Right Left	103 ± 21 103 ± 20	9.3 8.9	0.97 (0.94–0.99) 0.96 (0.93–0.98)	4.8	0.990
	CR (%)	Right Left	69.3 ± 5.4 69.2 ± 4.9	3.5 3.5	0.93 (0.86–0.96) 0.93 (0.90–0.98)	2.2	0.826
	FR (%)	Right Left	30.7 ± 5.4 30.8 ± 5.0	8.1 7.9	0.83 (0.70–0.91) 0.86 (0.63–0.96)	5.2	0.888

SD: Standard Deviation; ES: Effect Size; CV: Coefficient of Variation; BA: Bilateral Asymmetry; SF: Step Frequency; SL: Step Length; CT: Contact Time; FT: Flight Time; CR: Contact Ratio; FR: Flight Ratio.

**Table 3:** Pairwise comparisons between mean values at 12 and 14 km·h^−1^ for: coefficients of variation, percentages of bilateral asymmetry and mean values of all variables studied.

Variables	12 km·h^−1^	14 km·h^−1^	12 vs. 14 km·h^−1^ (*p*-Value (ES))		
			*p*-Value (ES)	CV	BA
SF (spm)	177.5 ± 7.3	181.5 ± 8.5	0.013 * (0.6)	0.177 (0.3)	0.592 (0.3)
SL (cm)	111.8 ± 5.0	127.3 ± 6.3	<0.001 ** (3.0)	0.349 (0.2)	0.797 (0.1)
CT (ms)	249 ± 16	229 ± 14	<0.001 ** (1.3)	0.928 (0.0)	0.367 (0.2)
FT (ms)	91 ± 21	103 ± 21	<0.001 ** (0.5)	0.003 * (0.4)	0.817 (0.0)
CR (%)	73.4 ± 5.6	69.3 ± 5.2	<0.001 ** (0.7)	0.576 (0.1)	0.786 (0.1)
FR (%)	26.7 ± 5.6	30.7 ± 5.3	<0.001 ** (0.7)	0.002 * (0.6)	0.497 (0.2)

* *p* < 0.05; ** *p* < 0.001; ES: Effect Size; CV: Coefficient of Variation; BA: Bilateral Asymmetry; SF: Step Frequency; SL: Step Length; CR: Contact Ratio; FR: Flight Ratio.

## Data Availability

The data presented in this study are available on request from the corresponding author due to the data is unavailable due to privacy or ethical restrictions.

## Supplementary Materials

The following supporting information can be downloaded at: https://www.mdpi.com/article/10.3390/sports12050117/s1, Table S1: Summary results of all running variables reported by the RunScribe system. Mean values (right and left), reliability data and mean bilateral asymmetry (%) for each kinematic variable at 12 and 14 km·h^−1^. Results of inter-limb paired *t*-tests for all parameters of interest at both speeds.
