# Supplementary material for: Bilateral Asymmetry of Spatiotemporal Running Gait Parameters in U14 Athletes at Different Speeds
Source: Sports (Basel). 2024 Apr 27;12(5):117. doi: 10.3390/sports12050117 (PMC11125289; doi:10.3390/sports12050117)
Supplement: Supplementary file 1 [file sports-12-00117-s001.zip › sports-2959521-supplementary.pdf]

Supplementary Materials:

Table S1: Summary results of all running variables reported by the RunScribe system. Mean values (right and left), reliability data and mean bilateral asymmetry (%) for each kinematic variable at 12 and 14 km·h^{-1}. Results of inter-limb paired t-tests for all parameters of interest at both speeds.

**Variables**	**Leg**	**12km·h^{-1}**				**14km·h^{-1}**			
		Mean (SD)	*CV (%)*	*BA (%)*	*p-value (ES)*	*Mean (SD)*	*CV (%)*	*BA (%)*	*p-value*
LSS (kN.m^{-1})	Right	7.6 ± 1.3	7.5	4.8	0.368	7.6 ± 1.3	10.7	5.7	0.888
	Left	7.6 ± 1.4	7.6			7.6 ± 1.2	7.9		
VSS (kN.m^{-1})	Right	16.4 ± 1.3	3.4	1.5	0.706	17.8 ± 1.5	3.6	1.9	0.648
	Left	16.3 ± 1.4	3.1			17.8 ± 1.5	3.4		
FSP (a. u.)	Right	7.6 ± 3.0	21.8	13.4	0.790	7.4 ± 3.0	20.8	15.4	0.707
	Left	7.7 ± 2.7	20.3			7.3 ± 2.6	19.3		
SA (º)	Right	2.2 ± 0.9	20.4	10.8	0.307	2.4 ± 0.9	18.2	10.1	0.932
	Left	2.1 ± 0.9	21.2			2.4 ± 0.8	17.5		
PExc	Right	-14.3 ± 5.9	19.6	14.5	0.648	-15.7 ± 6.0	20.1	22.5	0.890
	Left	-13.9 ± 4.6	18.0			-15.6 ± 4.9	19.0		
MPV	Right	771 ± 233	19.1	20.4	**0.019*** (0.4)	951 ± 251	18.9	16.6	0.131
	Left	683 ± 186	20.9			893 ± 238	18.9		
$t_{\text{(FSàMPV)}}$	Right	19.0 ± 6.5	26.9	20.9	0.445	20.2 ± 6.4	25.6	18.7	0.451
	Left	18.1 ± 5.5	28.2			19.5 ± 6.5	28.5		
$t_{\text{(MPVàMP)}}$	Right	33.1 ± 9.7	22.4	18.2	0.183	28.3 ± 7.8	27.0	17.7	0.577
	Left	35.0 ± 9.9	23.0			29.0 ± 7.2	23.4		
$t_{\text{(MPàTO)}}$	Right	195.4 ± 19.0	4.8	2.9	0.748	180.6 ± 16.5	4.9	2.8	0.869
	Left	195.0 ± 17.3	4.9			180.4 ± 13.7	4.4		
Shock	Right	14.7 ± 1.3	9.7	5.6	0.520	15.1 ± 1.6	10.0	7.2	0.333
	Left	14.8 ± 1.5	9.9			15.4 ± 1.6	10.2		
Impact	Right	9.4 ± 2.9	22.1	12.6	0.122	9.8 ± 3.3	20.5	17.2	0.136
	Left	9.8 ± 3.1	22.2			10.5 ± 3.1	19.7		
Braking	Right	10.6 ± 1.9	15.8	8.0	0.372	10.8 ± 1.8	16.5	9.0	0.500
	Left	10.4 ± 1.6	16.2			10.6 ± 1.5	18.6		
Peak VGRF	Right	3.5 ± 0.4	5.4	3.2	0.375	3.8 ± 0.5	5.5	3.3	0.863
	Left	3.5 ± 0.4	5.5			3.8 ± 0.4	5.3		
VGRF Rate	Right	39.9 ± 2.7	3.0	2.1	0.478	43.0 ± 2.8	3.0	2.2	0.790
	Left	39.7 ± 2.7	3.0			43.0 ± 2.5	3.0		
HGRF Rate	Right	7.3 ± 0.4	3.8	1.6	0.248	8.6 ± 0.4	3.9	1.4	0.569
	Left	7.3 ± 0.4	4.2			8.6 ± 0.4	4.0		
Total F Rate	Right	107.4 ± 4.0	2.7	1.0	0.206	121.2 ± 4.9	2.9	0.9	0.456
	Left	107.7 ± 4.1	2.9			121.0 ± 4.8	3.0		

*$p<0.05$; SD: Standard deviation;.ES: Effect size; CV: Coefficient of variation; MDC: Minimum detectable change; SF: Step frequency; SL: Step length; CR: Contact ratio; FR: Flight ratio; LSS: Leg spring stiffness; VSS: Vertical Spring Stiffness; FSP: Foot strike pattern; SA: Stride angle; Pr. Ex.: Pronation excursion; MPV: Maximum pronation velocity; $t_{\text{(FSàMPV)}}$: time from foot strike to maximum pronation velocity; $t_{\text{ MPVàMP)}}$: time from maximum pronation velocity to maximum pronation; $t_{\text{(MPàTO)}}$: time from maximum pronation to toe off; VGRF: Vertical ground reaction force; HGRF: Horizontal ground reaction force; F: Force.
